# Analytic solutions of variance swaps for Heston models with stochastic long-run mean of variance and jumps

**DOI:** 10.1371/journal.pone.0318886

**Published:** 2025-03-25

**Authors:** Jing Fu

**Affiliations:** 1 School of Mathematics, Southwestern University of Finance and Economics, Chengdu, China; Cyprus International University Faculty of Engineering: Uluslararasi Kibris Universitesi Muhendislik Fakultesi, TÜRKIYE

## Abstract

This paper presents the pricing formulas for variance swaps within the Heston model that incorporates jumps and a stochastic long-term mean for the underlying asset. By leveraging the Feynman-Kac theorem, we derive a partial integro-differential equation (PIDE) to obtain the joint moment-generating function for the aforementioned model. Furthermore, we provide a series pricing formula for discretely sampled variance swap, derived through the use of this joint moment-generating function. Additionally, we discuss the limiting properties of the pricing formula for discretely sampled variance swap, namely, the pricing formula for continuously sampled variance swap. Finally, to demonstrate the efficacy of the pricing formula, we conduct several numerical simulation experiments, including comparisons with Monte Carlo (MC) simulation results and an analysis of the impact of parameter variations on the strike price of variance swaps.

## 1 Introduction

Currently, as financial markets become increasingly complex, volatility risk has garnered significant attention in the financial industry. In order to effectively address this risk, volatility derivatives have become essential tools for measuring and managing financial risk. With the sharp increase in trading volumes, the precise and efficient determination of volatility derivative prices has emerged as a central topic in quantitative finance and risk management.

In 1973, the classic option pricing model proposed by Black and Scholes [1] and Merton [2] assumed constant asset price volatility. However, empirical studies have shown that the implied volatility reverse-engineered from the Black-Scholes formula is often non-constant, exhibiting the “volatility smile” phenomenon. This suggests that stock price volatility may be related to its level, time, or external random factors. As a result, many researchers have turned to studying stochastic volatility models, with the Stochastic Volatility (SV) model receiving particular attention.

Subsequently, Hull and White [3] introduced a continuous-time stochastic volatility model and derived a European option pricing formula via a second-order Taylor expansion. Around the same time, Scott [4] and Stein [5] presented modeling volatility using an Ornstein-Uhlenbeck process with mean-reverting characteristics and provided closed-form pricing formulas. However, their model could not prevent volatility from becoming negative, and the assumption of zero correlation between the underlying asset price and volatility was somewhat unrealistic. A few years later, Schöbel and Zhu [6] extended a model allowing for correlation between the underlying stock returns and instantaneous volatility, obtaining a closed-form solution for the European option pricing formula through an inverse Fourier transform method.

Furthermore, Heston [7] modeled volatility using the CIR model and derived a closed-form solution for the standard European option pricing through affine structure and Fourier transform methods. This affine stochastic volatility model not only provides a closed-form pricing formula but also adeptly captures the phenomenon of the “volatility smile”, making it a fundamental model for pricing volatility derivatives and instigating widespread research efforts. For instance, Elliott and Lian [8] studied the pricing of variance and volatility swaps under discrete observations using a segmented Heston stochastic volatility model, obtaining accurate closed-form solutions. He and Zhu [9] developed a hybrid model combining CIR random interest rates and Heston stochastic volatility to analyze the pricing formula for variance and volatility swaps, demonstrating convergence and verifying the accuracy of swap prices. Additionally, Kim and Kim [10] addressed the pricing issue of generalized variance swaps within the Heston-CIR hybrid model, offering exact solutions for the fair strike prices of these swaps. He and Lin [11] introduced a three-factor model incorporating random volatility and interest rates, deriving an exact analytical pricing formula for foreign exchange options. Najafi and Mehrdoust [12] analyzed the pricing of European options with proportional transaction costs in an incomplete market setting using two long-memory versions of the Heston model. However, the Heston model does have certain limitations, for example, the square root specification is typically not suitable for modeling index returns [13,14]. Additionally, Bakshi et al. [15] pointed out that the volatility process often exhibits non-linear mean-reverting characteristics.

Considering that the constant long-term average variance in the Heston model fails to capture the temporal market volatility dynamics, Byelkina and Levin [16] and Forde and Jacquier [17] suggested introducing a time-dependent mean-reverting variance level to better capture the term structure of implied volatility and variance swap curves. He and Chen [18] proposed a novel stochastic volatility model where the long-term mean of volatility in the Heston model itself follows a stochastic process, deriving a closed-form pricing formula for European options within this framework. Their empirical study, employing adaptive sample annealing, demonstrated the superiority of this pricing model over the Heston model. Yoon et al. [19] further extended the long-term variance mean of the Heston model to a stochastic process with mean-reverting characteristics and derived fair strike prices for variance swaps under this setup. Experimental results indicate that the stochastic long-run mean of variance plays a significant role in determining the fair strike prices of variance swaps in turbulent market.

Meanwhile, financial markets are often subject to sharp price fluctuations due to external shocks, such as major political events, economic data releases, or natural disasters, which continuous asset pricing models may not fully capture. To better represent asset price dynamics, researchers have incorporated jump risks into continuous models. For instance, Zheng and Kwok [20] derived pricing formulas for generalized variance swaps within a stochastic volatility framework where both the asset price and variance process allow for simultaneous jumps. Cui et al. [21] utilized frame duality and density projection method, combined with a novel weak approximation scheme based on continuous-time Markov chain (CTMC), to provide pricing formulas for volatility derivatives under discrete sampling. Wu et al. [22] proposed a variance swap valuation model that combines multi-factor stochastic spot variance and long-term variance, while allowing for mean reversion in asset prices and a co-jump structure. Empirical results demonstrate that this model outperforms other models. Wang and Guo [23] analyzed the pricing of variance and volatility swaps within the framework of a double Heston jump-diffusion model with approximate fractional stochastic volatility. Additionally, Ascione et al. [24] combined the Heston-CIR model with a Lévy process to enhance pricing accuracy in the foreign exchange (FX) market, presenting a new formula that more closely aligns with the observed price distribution.

The main goal of this paper is to propose a novel stochastic volatility model that includes jump processes and a stochastic long-term mean for variance, and to investigate the pricing of variance swaps within this framework. This model, which integrates asset price jumps and a stochastic long-term mean for variance, is more comprehensive than existing models.

The rest of this paper is organized as follows: Sect 2 introduces the new model and obtains its joint moment generating function (MGF) in a power series form. Sect 3 utilizes the joint MGF derived in Sect 2 to develop pricing formulas for variance swaps under both discrete and continuous sampling. Sect 4 show cases numerical experiments and examples to demonstrate the importance of incorporating a stochastic long-term mean. The concluding section offers final remarks.

## 2 The newly proposed model and its MGF

In this section, we aim to propose a new model based on the Yoon et al. [19]’s model , and derive the joint moment generating function(MGF) for this newly models. Consider the risk-neutral probability measurable space $\left(\Omega ,F_{t},\mathbb{Q}\right)$ in which we give the newly models as follows


$$\begin{array}{c} \{\begin{array}{c} \frac{dS_{t}}{S_{t}}=(r-d-\lambda m)dt+{\sqrt{v}}_{t}dW_{t}^{S}+(e^{J^{S}}-1)dN_{t},\\ dv_{t}=\kappa_{v}(\theta_{t}-v_{t})dt+\sigma_{v}{\sqrt{v}}_{t}dW_{t}^{v}+J^{v}dN_{t},\\ d\theta_{t}=\kappa_{\theta }(\tilde{\theta }-\theta_{t})dt+\sigma_{\theta }dW_{t}^{\theta }, \end{array} \end{array}$$ (1)

where *r*, *d*, *κ_v_*, *κ_θ_*, $\tilde{\theta }$, *σ_v_* and *σ_θ_* are positive constants, *λ* denotes the jump intensity and $m=E^{\mathbb{Q}}[e^{J^{S}}\;-\;1]$ represents the average jump amplitude of the price. Moreover, $W_{t}^{S}$, $W_{t}^{v}$ and $W_{t}^{\theta }$ are standard Brownian motions with $<dW_{t}^{S},dW_{t}^{v}>=\rho_{sv}dt$ and $<dW_{t}^{S},dW_{t}^{\theta }>=<dW_{t}^{v},dW_{t}^{\theta }>=0$, *N_t_* is independent with $W_{t}^{S}$ and $W_{t}^{v}$. *J^S^* and *J^v^* represent the jump sizes of the price and variance, respectively, assumed to be independent of $W_{t}^{S}$, $W_{t}^{v}$, and *N_t_*. For easy of solving the analytic solutions, we further assume that


$$J^{v}∼\exp\left(\frac{1}{\eta }\right),\;\;J^{S}|J^{v}∼\text{ N}\left(v+\rho_{J}J^{v},\delta^{2}\right).$$

Note that the proposed new model (1) encompasses several well-known models as special cases. The first is that *θ_t_*=*θ* a constant, it is reduced to the case considered in Zheng and Kwok [20]. The second is that no jump diffusion occurs in model (1) , it is reduced to the case considered in Yoon et al. [19]. The third is that both *θ_t_* a constant and no jump diffusion occurs in model (1) , it is reduced to the classic Heston stochastic volatility model. For convenience, we define xt= ln ⁡ St. To achieve the analytic solutions for variance swaps to the new model (1), we end this section by providing the expression for the joint moment generating function (MGF) of the joint process *x_t_*, *v_t_*, and *θ_t_* shown as follows.

**2.1.** *Assume that the dynamics of the asset price* *S_t_* *follows dynamics and denote*


$$U\left(x_{t},v_{t},\theta_{t},t\right)=E^{\mathbb{Q}}\left[e^{\phi x_{T}+bv_{T}+c\theta_{T}+\gamma }|F_{t}\right],$$

*Then we have*


$$\begin{array}{c} U\left(x_{t},v_{t},\theta_{t},t\right)=e^{\phi x_{t}+B(q,\tau )v_{t}+C(q,\tau )\theta_{t}+D(q,\tau )+E(q,\tau )}, \end{array}$$ (2)

*where* *τ* = *T* − *t* *and*


B(q,τ)=1σv2 [ (κv−ρsvσvϕ)−δ1⋅sinh ⁡  (δ12τ)+δ2 cosh ⁡  (δ12τ)cosh ⁡  (δ12τ)+δ2 sinh ⁡  (δ12τ)],C(q,τ)=−κvδ1e−κθτσv2 [∑n=0∞(−u)n (e (κθ−nδ1)τ−1κθ−nδ1−u (e (κθ−(n+1)δ1)τ−1)κθ−(n+1)δ1)]+κv (κv−ρsvσvϕ)κθσv2 (1−e−κθτ)+ce−κθτ,

D(q,τ)=(r−d)ϕτ+θ~κvσv2 [(κv−ρsvσvϕ)τ−2ln ⁡  (cosh ⁡  (δ12τ)+δ2 sinh ⁡  (δ12τ)) ]+κv2σθ2 (κv−ρsvσvϕ)2κθ2σv4[ (κv−ρsvσvϕ)τ−2ln ⁡  (cosh ⁡  (δ12τ)+δ2 sinh ⁡  (δ12τ))]− (κvσθ2 (κv−ρsvσvϕ)2κθ2σv2+θ~) (C(q,τ)−c)−κvσθ24κθ (C(q,τ)2−c2)+κv2σθ2δ1 (κv−ρsvσvϕ)2κθ2σv4[−2δ1 ln ⁡  (cosh ⁡  (δ12τ)+δ2 sinh ⁡  (δ12τ))+ ∑i=0∞(−u)i (e− (κθ+iδ1)τ−1− (κθ+iδ1)−u⋅e− (κθ+(i+1)δ1)τ−1− (κθ+(i+1)δ1))]−κvσθ2δ1c2κθσv2 [∑i=0∞(−u)i (e− (κθ+iδ1)−1− (κθ+iδ1)−ue− (κθ+(i+1)δ1)τ−1− (κθ+(i+1)δ1))]+κv2δ12σθ22κθσv4 [τκθ+e−κθτ−1κθ2+ ∑i=1∞(−u)iκθ−iδ1 (e−iδ1τ−1−iδ1−e−κθδ1τ−1−κθ)+∑i=0∞(−u)i+1κθ−(i+1)δ1 (e−(i+1)δ1τ−1−(i+1)δ1−e−κθδ1τ−1−κθ)+∑n=1∞∑i=0∞(−u)n+iκθ−iδ1 (e−(n+i)δ1τ−1−(n+i)δ1−e−(κθ+nδ1)τ−1−(κθ+nδ1))+∑n=1∞∑i=0∞(−u)n+i+1κθ−(i+1)δ1 (e−(n+i+1)δ1τ−1−(n+i+1)δ1−e−(κθ+nδ1)τ−1−(κθ+nδ1))+∑n=0∞∑i=0∞(−u)n+i+1κθ−iδ1 (e−(n+i+1)δ1τ−1−(n+i+1)δ1−e−(κθ+(n+1)δ1)τ−1−(κθ+(n+1)δ1))+∑n=0∞∑i=0∞(−u)n+i+2κθ−(i+1)δ1 (e−(n+i+2)δ1τ−1−(n+i+2)δ1−e−(κθ+(n+1)δ1)τ−1−(κθ+(n+1)δ1))],E(q,τ)=−λ(mϕ+1)τ+λσv2eϕv+ϕ2δ122m1+ηδ1τ−λσv2eϕv+ϕ2δ222ηm12−η2δ12 ln ⁡ m1+ηδ1+ (m1u−ηδ1u)e−δ1τm1+ηδ1+ (m1u−ηδ1u),

with $q={\left(\phi ,b,c,r\right)}^{T}$ and

$$u=\frac{1-\delta_{2}}{1+\delta_{2}},\;\;m_{1}=\sigma_{v}^{2}-\eta \left(\kappa_{v}-\rho_{sv}\sigma_{v}\phi +\sigma_{v}^{2}\phi \rho_{J}\right),$$

with


$$\begin{array}{c} \delta_{1}=\sqrt{{\left(\kappa_{v}-\rho_{sv}\sigma_{v}\phi \right)}^{2}+\sigma_{v}^{2}\left(\phi -\phi^{2}\right)},\;\;\delta_{2}=\frac{\left(\kappa_{v}-\rho_{sv}\sigma_{v}\phi \right)-b\sigma_{v}^{2}}{\delta_{1}}. \end{array}$$

**Proof.** By using Feynman-Kac theorem, we obtain $U\left(x_{t},v_{t},\theta_{t},t\right)$ satisfies the following PIDE equation


$$\begin{array}{c} \begin{array}{ccc} \frac{\partial U}{\partial \tau } & =\left(r-d-\lambda m-\frac{v}{2}\right)\frac{\partial U}{\partial x}+\kappa_{v}(\theta -v)\frac{\partial U}{\partial v}+\kappa_{\theta }\left(\tilde{\theta }-\theta \right)\frac{\partial U}{\partial \theta } & \\ & +\frac{v}{2}\frac{\partial^{2}U}{\partial x^{2}}+\frac{\sigma_{v}^{2}}{2}v\frac{\partial^{2}U}{\partial v^{2}}+\frac{\sigma_{\theta }^{2}}{2}\frac{\partial^{2}U}{\partial \theta^{2}}+\rho_{sv}\sigma_{v}v\frac{\partial^{2}U}{\partial x\partial v}\\ & +\lambda E^{\mathbb{Q}}\left[U\left(x+J^{S},v+J^{v},\theta ,\tau \right)-U(x,v,\theta ,\tau )\right], \end{array} \end{array}$$ (3)

with $U{|}_{\tau =0}=e^{\phi x_{T}+bv_{T}+c\theta_{T}+\gamma }$. Since has affine structure, we guess that $U\left(x_{t},v_{t},\theta_{t},t\right)$ admits an analytic solution with form given by


$$\begin{array}{c} U\left(x_{t},v_{t},\theta_{t},\tau \right)=e^{\phi x_{t}+B(q,\tau )v_{t}+C(q,\tau )\theta_{t}+D(q,\tau )+E(q,\tau )}. \end{array}$$ (4)

By putting into , it gives


$$\begin{array}{ccc} v\frac{\partial B}{\partial \tau }+\theta \frac{\partial C}{\partial \tau }+\frac{\partial D}{\partial \tau }+\frac{\partial E}{\partial \tau } & =\left(r-d-\lambda m-\frac{v}{2}\right)\phi +\kappa_{v}(\theta -v)B+\kappa_{\theta }(\tilde{\theta }-\theta )C+\frac{v}{2}\phi^{2} & \\ & +\frac{\sigma_{v}^{2}}{2}vB^{2}+\frac{\sigma_{\theta }^{2}}{2}C^{2}+\rho_{sv}\sigma_{v}v\phi B+\lambda E^{\mathbb{Q}}\left[e^{\phi J^{S}+BJ^{v}}-1\right], \end{array}$$

which by separating variables can be divided into four ODEs


$$\begin{array}{c} \{\begin{array}{c} \frac{\partial B}{\partial \tau }=-\frac{1}{2}\left(\phi -\phi^{2}\right)-\left(\kappa_{v}-\rho_{sv}\sigma_{v}\phi \right)B+\frac{\sigma_{v}^{2}}{2}B^{2},\\ \frac{\partial C}{\partial \tau }=\kappa_{v}B-\kappa_{\theta }C,\\ \frac{\partial D}{\partial \tau }=(r-d)\phi +\kappa_{\theta }\tilde{\theta }C+\frac{\sigma_{\theta }^{2}}{2}C^{2},\\ \frac{\partial E}{\partial \tau }=\lambda \left(E^{\mathbb{Q}}\left[e^{\phi J^{S}+BJ^{v}}-1\right]-m\phi \right), \end{array} \end{array}$$ (5)

with initial conditions


B(q,0)=b,C(q,0)=c,D(q,0)=γ,E(q,0)=0.

Obviously, the ODE governing *B* ( *ϕ* , *τ* )  is a Riccati equation with constant coefficients, which is not difficult to solve. Thus, the details are omitted.

By integrating both sides of the ODE governing *C* ( *ϕ* , *τ* )  and assuming


$$\frac{1}{1+ue^{-\delta_{1}\tau }}=\overset{\infty }{\underset{n=0}{\sum }}{\left(-u\right)}^{n}e^{-n\delta_{1}\tau },\;|u|<1.$$

The expression of *C* ( *ϕ* , *τ* )  can be easily worked out, one has


$$\begin{array}{ccc} C(q,\tau ) & =-\frac{\kappa_{v}\delta_{1}e^{-\kappa_{\theta }\tau }}{\sigma_{v}^{2}}\left[\overset{\infty }{\underset{n=0}{\sum }}{\left(-u\right)}^{n}\left(\frac{e^{\left(\kappa_{\theta }-n\delta_{1}\right)\tau }-1}{\kappa_{\theta }-n\delta_{1}}-u\frac{\left(e^{\left(\kappa_{\theta }-(n+1)\delta_{1}\right)\tau }-1\right)}{\kappa_{\theta }-(n+1)\delta_{1}}\right)\right] & \\ & +\frac{\kappa_{v}\left(\kappa_{v}-\rho_{sv}\sigma_{v}\phi \right)}{\kappa_{\theta }\sigma_{v}^{2}}\left(1-e^{-\kappa_{\theta }\tau }\right)+ce^{-\kappa_{\theta }\tau }, \end{array}$$

Furthermore,


$$\begin{array}{c} D(q,\tau )=(r-d)\phi \tau +\kappa_{\theta }\tilde{\theta }\int_{0}^{\tau }C(q,s)ds+\int_{0}^{\tau }\frac{\sigma_{\theta }^{2}}{2}C^{2}(q,s)ds+\gamma . \end{array}$$ (6)

By exploiting the ODEs , we are able to obtain the relation


$$\begin{array}{c} \{\begin{array}{c} \kappa_{\theta }\int_{0}^{\tau }C(q,s)ds=\kappa_{v}\int_{0}^{\tau }B(q,s)ds-C(q,\tau )+c,\\ \int_{0}^{\tau }C^{2}(q,s)ds=\frac{1}{\kappa_{\theta }}\left[\kappa_{v}\int_{0}^{\tau }B(q,s)C(q,s)ds-\frac{1}{2}C^{2}(q,\tau )+\frac{1}{2}C^{2}\right]. \end{array} \end{array}$$ (7)

Applying to , *D* ( *q* , *τ* )  transforms into


$$\begin{array}{c} \begin{array}{ccc} D(q,\tau ) & =(r-d)\phi \tau +\tilde{\theta }\left[\kappa_{v}\int_{0}^{\tau }B(q,s)ds-C(q,\tau )+c\right] & \\ & +\frac{\sigma_{\theta }^{2}}{2\kappa_{\theta }}\left[\kappa_{v}\int_{0}^{\tau }B(q,s)C(q,s)ds-\frac{1}{2}C^{2}(q,\tau )+\frac{1}{2}C^{2}\right]+\gamma . \end{array} \end{array}$$ (8)

To calculate , we need to integrate *B* ( *q* , *s* )  and *B* ( *q* , *s* ) *C* ( *q* , *s* ) . Through direct calculation, the integral of *B* ( *q* , *s* )  yields


∫ 0τB(q,s)ds=1σv2 [ (κv−ρsvσvϕ)τ−2ln ⁡  (cosh ⁡  (δ12τ)+δ2 sinh ⁡  (δ12τ))], (9)

and the integral of *B* ( *q* , *s* ) *C* ( *q* , *s* )  can be computed as


κvσθ22κθ ∫ 0τB(q,s)C(q,s)ds=κv2σθ2 (κv−ρsvσvϕ)2κθ2σv4[ (κv−ρsvσvϕ)τ−2ln ⁡  (cosh ⁡  (δ12τ)+δ2 sinh ⁡  (δ12τ))]− (κvσθ2 (κv−ρsvσvϕ)2κθ2σv2) (C(q,τ)−c)+κv2σθ2δ1 (κv−ρsvσvϕ)2κθ2σv4[−2δ1 ln ⁡  (cosh ⁡  (δ12τ)+δ2 sinh ⁡  (δ12τ))+∑i=0∞(−u)i (e− (κθ+iδ1)τ−1− (κθ+iδ1)−u⋅e− (κθ+(i+1)δ1)τ−1− (κθ+(i+1)δ1))]−κvσθ2δ1c2κθσv2 [∑i=0∞(−u)i (e− (κθ+iδ1)−1− (κθ+iδ1)−ue− (κθ+(i+1)δ1)τ−1− (κθ+(i+1)δ1))]+κv2δ12σθ22κθσv4 [τκθ+e−κθτ−1κθ2+ ∑i=1∞(−u)iκθ−iδ1 (e−iδ1τ−1−iδ1−e−κθδ1τ−1−κθ)+∑i=0∞(−u)i+1κθ−(i+1)δ1 (e−(i+1)δ1τ−1−(i+1)δ1−e−κθδ1τ−1−κθ)+∑n=1∞∑i=0∞(−u)n+iκθ−iδ1 (e−(n+i)δ1τ−1−(n+i)δ1−e−(κθ+nδ1)τ−1−(κθ+nδ1))+∑n=1∞∑i=0∞(−u)n+i+1κθ−(i+1)δ1 (e−(n+i+1)δ1τ−1−(n+i+1)δ1−e−(κθ+nδ1)τ−1−(κθ+nδ1))+∑n=0∞∑i=0∞(−u)n+i+1κθ−iδ1 (e−(n+i+1)δ1τ−1−(n+i+1)δ1−e−(κθ+(n+1)δ1)τ−1−(κθ+(n+1)δ1))+∑n=0∞∑i=0∞(−u)n+i+2κθ−(i+1)δ1 (e−(n+i+2)δ1τ−1−(n+i+2)δ1−e−(κθ+(n+1)δ1)τ−1−(κθ+(n+1)δ1))]. (10)

Substituting and into yields the expression for *D* ( *q* , *τ* ) . Due to $J^{v}∼\exp\left(\frac{1}{\eta }\right),J^{S}|J^{v}∼\text{ N }\left(v+\rho_{J}J^{v},\delta^{2}\right)$, we have


$$\begin{array}{c} \begin{array}{ccc} \frac{\partial E}{\partial \tau } & =\lambda E^{\mathbb{Q}}\left[\left(e^{\phi J^{s}+BJ^{\nu }}-1\right)\right]-\lambda m\phi & \\ & =-\lambda (m\phi +1)+\lambda e^{\phi v+\frac{\delta^{2}\phi^{2}}{2}}\frac{1}{1-(\phi \rho_{J}B)\eta }. \end{array} \end{array}$$ (11)

Solving completes the proof. □

## 3 Analytic solutions for variance swaps

In this section, we introduce variance swaps and using the joint moment generating function to derive the pricing formulas for variance swaps under the frame of discrete and continuous sampling, respectively.

To do this, we first give the definitions of the annualized realized variance and volatility. According to Yuen et al. [25], the calculation of realized variance depends on whether it is based on the actual or log returns. The annualized realized variance of the actual returns and the log returns are represented as $RV_{var}^{\left(1\right)}$ and $RV_{var}^{\left(2\right)}$, respectively, which are given by


RVvar(1)=AFN∑i=1N (Sti−Sti−1Sti−1)2×1002,RVvar(2)=AFN∑i=1N (ln ⁡ StiSti−1)2×1002, (12)

where *t_i_*, *i* = 0 , 1 , ⋯ , *N* is the *i*-th observation time in  [ 0 , *T* ] , $S_{t_{i}}$ is the price of underlying asset at the *i*-th observation time *t_i_* and there are altogether *N* observations. *AF* is the annualized factor that converts this expression to an annualized variance. Let $\Delta_{t}=t_{i}-t_{i-1}=\frac{T}{N}$, the annualized factor $AF=\frac{1}{\Delta_{t}}=\frac{N}{T}$.

### 3.1 The discrete case

Variance swaps are special kinds of forward contracts. The price of variance swaps are called strike prices in the contracts. In this subsection, we derive the fair strike prices of variance swaps in terms of the joint moment generating function.

We denote $K_{var}^{\left(i\right)},i=1,2$ be the fair strike prices of variance swaps with realized variance $RV_{var}^{\left(i\right)},i=1,2$. The values of the variance at time *t* with maturity *T* can be presented as


$$\begin{array}{c} \begin{array}{c} V_{var}(t)=E^{\mathbb{Q}}[e^{-r(T-t)}(RV_{var}^{\left(i\right)}-K_{var}^{\left(i\right)})L|F_{t}],\;i=1,2. \end{array} \end{array}$$ (13)

where *L* is the nominal amount. To be fair to both parties, the value of the contract should be equal to zero when it is realized. Thus, the fair strike prices $K_{var}^{\left(i\right)},i=1,2$ are given by


Kvar(1)=Eℚ[RVvar(1)|F0]=AFN∑i=1NEℚ [ (Sti−Sti−1Sti−1)2 |F0]×1002,Kvar(2)=Eℚ[RVvar(2)|F0]=AFN∑i=1NEℚ [ (ln ⁡ StiSti−1)2 |F0]×1002. (14)

Since the expectation is additive, we use the additivity of expectations to calculate the fair delivery prices of variance swaps. Specifically, we first calculate the values of $E^{\mathbb{Q}}[{\left(\frac{S_{t_{i}}-S_{t_{i-1}}}{S_{t_{i-1}}}\right)}^{2}|F_{0}]$ and Eℚ [ (ln ⁡ StiSti−1)2 |F0 ]. Upon the introduction of variance swaps, we will illustrate the utilization of the joint moment-generating function to compute each term in the aforementioned summation. Consequently, the comprehensive pricing formulas for variance swaps with discrete sampling within the framework (1) are derived in the subsequent sections.

**Theorem 1.** *Let* $K_{var}^{\left(i\right)},i=1,2$ *be the fair delivery prices of discretely sampled variance swaps under the dynamics specified in* (1), *Then*,


$$\begin{array}{c} \begin{array}{ccc} K_{var}^{\left(1\right)} & =\frac{100^{2}AF}{N}\overset{N}{\underset{i=1}{\sum }}[e^{B(q_{2},t_{i-1},)v_{0}+C(q_{2},t_{i-1})\theta_{0}+D(q_{2},t_{i-1})+G(q_{2},t_{i-1})} & \\ & -2e^{B(q_{4},t_{i-1},)v_{0}+C(q_{4},t_{i-1})\theta_{0}+D(q_{4},t_{i-1})+G(q_{4},t_{i-1})}+1], \end{array} \end{array}$$ (15)

$$\begin{array}{c} \begin{array}{c} K_{var}^{\left(2\right)}=\frac{100^{2}AF}{N}\overset{N}{\underset{i=1}{\sum }}\frac{\partial^{2}}{\partial \phi^{2}}e^{B(q_{6};t_{i-1})v_{0}+C(q_{6},t_{i-1})\theta_{0}+D(q_{6},t_{i-1})+G(q_{6},t_{i-1})}{|}_{\phi =0}, \end{array} \end{array}$$ (16)

*where*


$$\begin{array}{c} \{\begin{array}{c} q_{1}={\left(2\;0\;0\;0\right)}^{⊤},\\ q_{2}={\left(0,B(q_{1},\tau ),C(q_{1},\tau ),D(q_{1},\tau )+G(q_{1},\tau )\right)}^{⊤},\\ q_{3}={\left(1\;0\;0\;0\right)}^{⊤},\\ q_{4}={\left(0,B(q_{3},\tau ),C(q_{3},\tau ),D(q_{3},\tau )+G(q_{3},\tau )\right)}^{⊤},\\ q_{5}={\left(\phi \;0\;0\;0\right)}^{⊤},\\ q_{6}={\left(0,B(q_{4},\tau ),C(q_{4},\tau ),D(q_{4},\tau )+G(q_{4},\tau )\right)}^{⊤}. \end{array} \end{array}$$ (17)

**Proof.** (i) Actual rate of return. Since


$$K_{var}^{\left(1\right)}=\frac{AF}{N}\overset{N}{\underset{i=1}{\sum }}E^{\mathbb{Q}}[{\left(\frac{S_{t_{i}}-S_{t_{i-1}}}{S_{t_{i-1}}}\right)}^{2}|F_{0}]\times 100^{2},$$

we only calculate the expression of $E^{\mathbb{Q}}[{\left(\frac{S_{t_{i}}-S_{t_{i-1}}}{S_{t_{i-1}}}\right)}^{2}|F_{t_{i-1}}]$.


$$\begin{array}{ll} & E^{\mathbb{Q}}[{\left(\frac{S_{t_{i}}-S_{t_{i-1}}}{S_{t_{i-1}}}\right)}^{2}|F_{0}]\;\\ =E^{\mathbb{Q}}\left[(e^{x_{t_{i}}-x_{t_{i-1}}}-1{)}^{2}|F_{0}\right]\\ =E^{\mathbb{Q}}\left[e^{2(x_{t_{i}}-x_{t_{i-1}})}|F_{0}\right]-2E^{\mathbb{Q}}\left[e^{\left(x_{t_{i}}-x_{t_{i-1}}\right)}|F_{0}\right]+1\;\\ =E^{\mathbb{Q}}[E^{\mathbb{Q}}[e^{2x_{t_{i}}}|F_{t_{i-1}}]e^{-2x_{t_{i-1}}}|F_{0}]\;\\ -2E^{\mathbb{Q}}[E^{\mathbb{Q}}[e^{x_{t_{i}}}|F_{t_{i-1}}]e^{-x_{t_{i-1}}}|F_{0}]+1\;\\ & =E^{\mathbb{Q}}\left[e^{B(q_{1},\tau )v_{t_{i-1}}+C(q_{1},\tau )\theta_{t_{i-1}}+D(q_{1},\tau )+E(q_{1},\tau )}|F_{0}\right]\\ & -2E^{\mathbb{Q}}\left[e^{B(q_{3},\tau )v_{t_{i-1}}+C(q_{3},\tau )\theta_{t_{i-1}}+D(q_{3},\tau )+E(q_{3},\tau )}|F_{0}\right]+1\;\\ & =e^{B(q_{2},t_{i-1},)v_{0}+C(q_{2},t_{i-1})\theta_{0}+D(q_{2},t_{i-1})+G(q_{2},t_{i-1})}\;\\ & -2e^{B(q_{4},t_{i-1},)v_{0}+C(q_{4},t_{i-1})\theta_{0}+D(q_{4},t_{i-1})+G(q_{4},t_{i-1})}+1,\; \end{array}$$

where $q_{1},\;q_{2},\;q_{3}$ and *q*_4_ are given by . Then the delivery price of discretely sampled variance swap is provided as


$$\begin{array}{l} \begin{array}{ccc} K_{var} & =\frac{100^{2}AF}{N}\overset{N}{\underset{i=1}{\sum }}[e^{B(q_{2},t_{i-1},)v_{0}+C(q_{2},t_{i-1})\theta_{0}+D(q_{2},t_{i-1})+G(q_{2},t_{i-1})} & \\ & -2e^{B(q_{4},t_{i-1},)v_{0}+C(q_{4},t_{i-1})\theta_{0}+D(q_{4},t_{i-1})+G(q_{4},t_{i-1})}+1]. \end{array} \end{array}$$

(ii) Log rate of return. Similar to $K_{var}^{\left(1\right)}$, we calculate the expression of Eℚ [ (ln ⁡ StiSti−1)2 |F0 ].


Eℚ [ (ln ⁡ StiSti−1)2 |F0 ]=Eℚ [(xti−xti−1)2 |F0]=Eℚ [∂2∂ϕ2eϕ(xti−xti−1) |F0 ] |ϕ=0=∂2∂ϕ2Eℚ [Eℚ [eϕxti |Fti−1 ]e−ϕxti−1 |F0] |ϕ=0=∂2∂ϕ2Eℚ [eB(q5,τ)vti−1+C(q5,τ)θti−1+D(q5,τ)+E(q5,τ) |F0] |ϕ=0=∂2∂ϕ2eB(q6;ti−1)v0+C(q6,ti−1)θ0+D(q6,ti−1)+G(q6,ti−1) |ϕ=0,

with *q*_5_ and *q*_6_ given by . As a result, the delivery price of discretely sampled variance swap is presented as


$$\begin{array}{c} \frac{100^{2}AF}{N}\overset{N}{\underset{i=1}{\sum }}\frac{\partial^{2}}{\partial \phi^{2}}e^{B(q_{6};t_{i-1})v_{0}+C(q_{6},t_{i-1})\theta_{0}+D(q_{6},t_{i-1})+G(q_{6},t_{i-1})}{|}_{\phi =0}. \end{array}$$

The proof is completed. □

The pricing formulas for the fair delivery prices of variance swaps under discrete sampling, as determined by , are predicated on finite observations. The following subsection investigates the limiting properties of these pricing formulas as *N* → + *∞* (i.e., *Δt* → 0).

### 3.2 The continuous case

In the previous section, we obtained the fair strike prices of variance swaps under discrete sampling. In this subsection, we will present the pricing formulas for variance swaps under continuous sampling.

The continuous model is widely used for variance swaps. According to [26], the realized variance consists of two parts: The first component accounts for the variance accumulated via the diffusion term in the asset price process, while the second component arises from the jumps in the asset price process. Therefore, the realized variance can be expressed as


RVcvar=lim ⁡ N→+∞1002T∑i=1N (Sti−Sti−1Sti−1)2=1002T [∫ 0Tvtdt+ ∑i=1NT(eJS−1)2]. (18)

As a result, the fair strike price *K_cvar_* of the continuously-sampled variance swap can be presented as


$$\begin{array}{c} \begin{array}{ccc} K_{cvar} & =E^{\mathbb{Q}}[RV_{cvar}|F_{0}] & \\ & =\frac{100^{2}}{T}\left[\int_{0}^{T}E^{\mathbb{Q}}[v_{t}|F_{0}]dt+E^{\mathbb{Q}}\left[\overset{N_{T}}{\underset{i=1}{\sum }}{\left(e^{J^{S}}-1\right)}^{2}\right]\right]. \end{array} \end{array}$$ (19)

**Theorem 2.** *The fair strike price* *K_cvar_* *of the continuously-sampled variance swap within the Heston model incorporating stochastic long-term variance mean and jumps can be determined as*


$$\begin{array}{c} \begin{array}{ccc} K_{cvar} & =\frac{100^{2}}{T}[\left(\tilde{\theta }+\frac{\lambda \eta }{\kappa_{v}}\right)T+\left(v_{0}-\tilde{\theta }-\frac{\kappa_{v}(\theta_{0}-\tilde{\theta })}{\kappa_{v}-\kappa_{\theta }}\right)\frac{1-e^{-\kappa_{v}T}}{\kappa_{v}} & \\ & +\frac{\kappa_{v}(\theta_{0}-\tilde{\theta })}{\kappa_{v}-\kappa_{\theta }}\frac{1-e^{-\kappa_{\theta }T}}{\kappa_{\theta }}+\lambda T\left(\frac{e^{2v+2\delta^{2}}}{1-2\rho_{J}\eta }-\frac{2e^{v+\delta^{2}∕2}}{1-\rho_{J}\eta }+1\right)]. \end{array} \end{array}$$ (20)

**Proof.** We know that *v_t_* and *θ_t_* satisfy


$$\begin{array}{c} \{\begin{array}{c} dv_{t}=\kappa_{v}(\theta_{t}-v_{t})dt+\sigma_{v}\sqrt{v_{t}}ddW_{t}^{v}+J^{v}dN_{t},\\ d\theta_{t}=\kappa_{\theta }(\tilde{\theta }-\theta_{t})dt+\sigma_{\theta }dW_{t}^{\theta }, \end{array} \end{array}$$ (21)

where $W_{t}^{v}$ and $W_{t}^{\theta }$ are two independent standard Brownian motions. Integrating both sides of , we have


$$\begin{array}{c} v_{t}-v_{0}=\kappa_{v}\int_{0}^{t}(\theta_{s}-v_{s})ds+\sigma_{v}\int_{0}^{t}\sqrt{v_{t}}dW_{s}^{v}+\overset{N_{T}}{\underset{i=1}{\sum }}J_{v}, \end{array}$$ (22)

$$\begin{array}{c} \theta_{t}-\theta_{0}=\kappa_{\theta }\int_{0}^{t}(\tilde{\theta }-\theta_{s})ds+\sigma_{\theta }\int_{0}^{t}dW_{s}^{\theta }. \end{array}$$ (23)

Taking the expectation on both sides of and , and then take the derivative with respect to *t*, one has


$$\begin{array}{rc} \frac{dE^{\mathbb{Q}}[v_{t}|F_{0}]}{dt}=\kappa_{v}(E^{\mathbb{Q}}[\theta_{t}|F_{0}]-E^{\mathbb{Q}}[v_{t}|F_{0}])+\lambda \eta , & \\ \frac{dE^{\mathbb{Q}}[\theta_{t}|F_{0}]}{dt}=\kappa_{\theta }(\tilde{\theta }-E^{\mathbb{Q}}[\theta_{t}|F_{0}]), & \end{array}$$

with initial conditions $E^{\mathbb{Q}}[v_{t}]{|}_{t=0}=v_{0}$ and $E^{\mathbb{Q}}[\theta_{t}]{|}_{t=0}=\theta_{0}$. By solving the above ODEs, $E^{\mathbb{Q}}[v_{t}]$ and $E^{\mathbb{Q}}[\theta_{t}]$ can be derived as


$$\begin{array}{c} \begin{array}{ccc} E^{\mathbb{Q}}[v_{t}|F_{0}] & =\tilde{\theta }(1-e^{-\kappa_{v}t})+\frac{\kappa_{v}(\theta_{0}-\tilde{\theta })}{\kappa_{v}-\kappa_{\theta }}(e^{-\kappa_{\theta }t}-e^{-\kappa_{v}t})+v_{0}e^{-\kappa_{v}t}+\frac{\lambda \eta }{\kappa_{v}}(1-e^{-\kappa_{v}t}), & \\ E^{\mathbb{Q}}[\theta_{t}|F_{0}] & =\tilde{\theta }+(\theta_{0}-\tilde{\theta })e^{-\kappa_{\theta }t}. \end{array} \end{array}$$ (24)

Since *J^S^* is independent of *N_t_*, we obtain


$$\begin{array}{c} \begin{array}{ccc} E^{\mathbb{Q}}\left[\overset{N_{T}}{\underset{i=1}{\sum }}{\left(e^{J^{S}}-1\right)}^{2}\right] & =\lambda TE^{\mathbb{Q}}\left[{\left(e^{J^{S}}-1\right)}^{2}\right] & \\ & =\lambda T\left(\frac{e^{2v+2\delta^{2}}}{1-2\rho_{J}\eta }-\frac{2e^{v+\delta^{2}∕2}}{1-\rho_{J}\eta }+1\right). \end{array} \end{array}$$ (25)

By substituting and into can be readily obtained, which complete the proof. □

## 4 Numerical examples

We present several numerical examples and compare the obtained conclusions with existing research findings. In our numerical examples, we employ two types of samples, labeled as Sample 1 and 2, where the model parameters are detailed in Table 1.

**Table 1:** Sample parameters of our model

Parameters	v_0_	*θ*_0_	*κ*_v_	*σ*_v_	*κ_θ_*	$\tilde{\theta }$	*σ_θ_*	*ρ*_sv_
**Sample 1**	0.1	0.15	5	0.005	4	0.1	0.01	-0.5
**Sample 2**	0.16	0.11	6.3	0.012	3.6	0.125	0.004	-0.7

Notes: The parameters *r*, *T*, *l*, *λ*, *η*, *v*, *ρ_J_* and *δ* are fixed at *r* = 0 . 01, *T* = 1, *l* = 15, *λ* = 0 . 2, *η* = 0 . 05, *v* = 0 . 01, $\rho_{J}=-0.086$ and *δ* = 0 . 02, respectively.

**Example 4.1.** *In this numerical experiment, we conduct a numerical comparison between the results derived from the pricing formulas and those obtained through Monte Carlo (MC) simulations. Given the similarity in pricing formulas for variance swaps based on actual returns and those based on logarithmic returns, we solely compare the prices of variance swaps based on actual returns with the outcomes of the Monte Carlo simulations. The Euler-Maruyama discretization for the Heston jump-diffusion model with a stochastic long-term jump mean can be expressed as follows*


$$\begin{array}{c} \begin{array}{c} \{\begin{array}{cc} S_{t_{i}} & =S_{t_{i-1}}e^{(r-\lambda m-\frac{1}{2}v_{t_{i-1}})\Delta t+\sqrt{v_{t_{i-1}}}\sqrt{\Delta t}W_{1,t_{i-1}}+\overset{N_{t_{i}}}{\underset{N_{t_{i-1}}}{\sum }}J^{S}},\\ v_{t_{i}} & =v_{t_{i-1}}+\kappa_{v}(\theta_{t_{i-1}}-v_{t_{i-1}})\Delta t+\overset{N_{t_{i}}}{\underset{N_{t_{i-1}}}{\sum }}J^{v}\\ & +\sigma_{v}\sqrt{v_{t_{i-1}}}\sqrt{\Delta t}(\rho_{sv}W_{1,t_{i-1}}+\sqrt{1-\rho_{sv}^{2}}W_{2,t_{i-1}}),\\ \theta_{t_{i}} & =\theta_{t_{i-1}}(\tilde{\theta }-\theta_{t_{i-1}})\Delta t+\sigma_{\theta }\sqrt{\Delta t}W_{3,t_{i-1}}, \end{array} \end{array} \end{array}$$ (26)

*where* $W_{1,t_{i-1}}$, $W_{2,t_{i-1}}$, $W_{3,t_{i-1}}$ *are three independent standard normal random variables.* *Δt* = *T* ∕ *N*. *J^S^* *and* *J^v^* *are random variables generated by normal distribution and exponential distribution, respectively.*

*We exhibit a comparison between the numerical implementation of and the Monte Carlo (MC) simulation results in S1 Fig and Table 2. S1 Fig displays three sets of data: one set represents the variance swap prices derived from 500,000 Monte Carlo simulations based on , another set is calculated using for discretely-sampled variance swaps, and the third set is obtained from for continuously-sampled variance swaps. In Table 2, we present a detailed listing of the numerical results for variance swaps under four different sampling frequencies along with their corresponding relative errors. In this table, AS denotes the discretely-sampled analytic solution, MC represents the Monte Carlo simulation results, CON stands for the continuously-sampled analytic solution, and RD signifies the standard error. Additionally, we have recorded the CPU runtime to facilitate a comprehensive comparison of the computational efficiency of the two methods. All calculations were performed on an Intel(R) Core(TM) i5-12500H processor. It is evident from the table that the analytic formula can produce highly accurate results within an extremely short period of time. Notably, as the sampling frequency increases, the time required to compute the fair strike price of variance swaps using Monte Carlo simulation becomes significantly longer than that required using our proposed analytic pricing formula. From S1 Fig and Table 2, we observe that as the sampling frequency increases, the strike price of the variance swap under discrete sampling gradually converges to that under continuous sampling. Furthermore, with increasing sampling frequency, the strike price of the variance swap under discrete sampling becomes very close to the price obtained through MC simulations, this indicates that the results obtained from our analytical results in Theorem 1 are in high agreement with those from the MC simulations.*

**Table 2:** Fair strike prices *K_var_* derived from MC simulation, the analytic formula and the continuous model, along with their corresponding relative errors.

Sample	*N*	AS	textbfMC	CON	RD
Sample 1	Monthly (*N* = 12)	1141.0764	1145.0146	1133.8657	0.3439
	CPU time (s)	0.0115	1.1463	-	-
	Fortnightly (*N* = 26)	1137.1822	1138.5908	1133.8657	0.1237
	CPU time (s)	0.0141	1.6675	-	-
	Weekly (*N* = 52)	1135.5212	1136.1883	1133.8657	0.0587
	CPU time (s)	0.01185	2.7977	-	-
	Daily (*N* = 252)	1134.2058	1134.3083	1133.8657	0.0090
	CPU time (s)	0.0147	11.4784	-	-
Sample 2	Monthly (*N* = 12)	1288.9977	1288.5884	1280.6165	-0.0318
	CPU time (s)	0.0071	1.1323	-	-
	Fortnightly (*N* = 26)	1284.4478	1283.9812	1280.6165	-0.0363
	CPU time (s)	0.0087	1.8132	-	-
	Weekly (*N* = 52)	1282.5236	1282.3325	1280.6165	-0.0149
	CPU time (s)	0.0092	3.6321	-	-
	Daily (*N* = 252)	1281.0086	1280.8717	1280.6165	-0.0107
	CPU time (s)	0.0136	10.6012	-	-

Notes: We provide numerical results for variance swaps under four different sampling frequencies, along with their corresponding relative errors. In this table, AS, MC, CON and RD represent the discretely-sampled analytic solution, Monte Carlo simulation results, continuously-sampled analytic solution and standard error, respectively.

**Example 4.2.** *By assigning different values to the parameters in system (1), our model encompasses the Heston model, the Heston model with simultaneous jumps, and the Heston model with stochastic long-run mean of variance. For example, if the parameters $\kappa_{\theta }=\sigma_{\theta }=\lambda =0$, our model deduce to the model framework in [27]. Using the parameters provided by [27], Table 3 presents the analytical solutions derived in this paper, the analytical solutions provided in [27], and the results obtained from Monte Carlo simulations. Clearly, Table 3 demonstrates that our pricing formula is consistent with the closed-form variance swap formula proposed by [27] using the single-factor Heston model. When the parameters satisfy $\kappa_{\theta }=\sigma_{\theta }=0$, our model reduces to the Heston model with simultaneous jumps, the system of equations obtained during the derivation of the joint moment generating function is consistent with [20]. Through computation, we obtain the same pricing formula as in [20] under this condition. On the other hand, when *λ* = 0, our model simplifies to the Heston model with a stochastic long-term variance mean proposed by [19], Table 4 presents the analytical solutions derived in this paper, the analytical solutions provided in [19], and the results obtained from Monte Carlo simulations.*

**Table 3:** Results of Heston model using parameters in [27]

Sampling frequency	MC	Zhu’s formula	Our formula
Monthly (*N* = 12)	243.2	242.7	242.71
Fortnightly (*N* = 26)	238.1	238.6	238.58
Weekly (*N* = 52)	237.4	237.1	237.11
Daily (*N* = 252)	236.5	236.1	236.08

Notes: The table presents the results of the Heston model using different sampling frequencies. The values are compared between MC simulation, Zhu’s formula, and our proposed formula.

**Table 4:** Results of our model using parameters in [19]

Sample	*N*	Our formula	Yoon’s formula	MC
Sample1	Monthly (*N*=12)	953.168	953.145	948.340
	Weekly (*N*=52)	950.843	950.838	948.411
	Daily (*N*=252)	950.248	950.248	951.106
Sample2	Monthly (*N*=12)	1272.507	1272.510	1276.980
	Weekly (*N*=52)	1267.881	1267.880	1265.570
	Daily (*N*=252)	1266.665	1266.670	1266.740
Sample3	Monthly (*N*=12)	775.506	775.506	773.783
	Weekly (*N*=52)	773.478	773.478	772.599
	Daily (*N*=252)	772.970	772.970	773.570

Notes: The table presents the results of our model using different sampling frequencies and samples. The values are compared between our formula, Yoon’s formula, and MC simulations.

**Example 4.3.** *We investigated the impact of key parameters *κ_θ_*, $\tilde{\theta }$, *σ_θ_*, and *θ*_0_ of the stochastic long-term mean on the strike price of variance swaps, while keeping all other parameters in the model consistent with those in Sample 2.*

*From S2 Fig, we observe that the primary parameters of the stochastic long-term mean significantly impact the strike price of the variance swap. Specifically, the strike price of the variance swap increases with an increase in *κ_θ_*. This is because *κ_θ_* determines the speed at which the long-term mean *θ_t_* reverts to its long-term average $\tilde{\theta }$. Specifically, when *κ_θ_* is high, *θ_t_* converges to $\tilde{\theta }$ more rapidly. Over the life of the contract, this leads to an increase in the variability of *v_t_*, which in turn amplifies the fluctuations in realized returns, thereby raising the fair strike price of the variance swap. Conversely, when *κ_θ_* is low, the reversion of *θ_t_* to $\tilde{\theta }$ occurs more slowly. Over the contract period, the variability of *v_t_* becomes relatively smaller, resulting in reduced fluctuations in realized returns and, consequently, a lower fair strike price for the variance swap. The strike price of the variance swap exhibits an increasing trend with an increase in $\tilde{\theta }$ and *θ*_0_, and the increase occurs at a relatively steady rate. This is because when $\tilde{\theta }$ is high, *θ_t_* tends to fluctuate around a higher level, thereby pulling *v_t_* toward higher values. This increases the variability of $\frac{S_{t_{i}}-S_{t_{i-1}}}{S_{t_{i}}}$, leading to a higher realized variance and, consequently, a higher fair strike price. In markets with low volatility expectations (e.g., during periods of economic stability or calm market sentiment), investors demand lower risk premiums. As a result, the fair strike price of variance swaps decreases. Additionally, if the market’s expectation of future volatility is high at the initial time, the volatility of asset prices will be greater in the early stages of the contract, resulting in higher realized variance and, consequently, an increase in the fair strike price of the variance swap. Furthermore, the strike price of the variance swap increases with an increase in *σ_θ_*, and the rate of increase gradually decreases with an increase in the sampling frequency. The increase in *σ_θ_* leads to a higher strike price for variance swaps due to the greater uncertainty and instability it introduces into the long-term volatility dynamics. This uncertainty increases the risk premium demanded by market participants, driving up the price of variance swaps. However, as sampling frequency increases, the rate at which the strike price rises slows down, reflecting the averaging effect of more frequent measurements.*

**Example 4.4.** *We further investigated the impact of key jump parameters *λ*, *η*, *v*, and *δ* on the strike price of variance swaps, while keeping all other parameter settings in the model consistent with those in Sample 2.*

*Observing S3 Fig reveals that the key parameters related to the jump component play a crucial role in determining the strike price of the variance swap. Specifically, the variance swap shows a high sensitivity to *λ*, with the strike price gradually increasing as *λ* increases. Specifically, the jump intensity *λ* reflects the probability of price jumps in the underlying asset within a unit of time. A higher jump intensity implies a greater likelihood of significant short-term fluctuations in the underlying asset price, indicating an expected increase in future volatility, leading to an increase in the price of the variance swap. A higher *λ* increases the uncertainty about future asset price movements. Investors and hedgers demand greater compensation for this heightened uncertainty, thereby leading to an increase in the fair strike price of the variance swap. The strike price of the variance swap gradually increases with an increase in *η*. This is because an increase in *η* will increase the volatility of the variance, leading to an increase in the volatility of the underlying asset price, thereby increasing the strike price of the variance swap. This increased variability in volatility translates to higher uncertainty about future market conditions. As a result, the strike price of the variance swap rises to reflect the heightened risk. The strike price of the variance swap shows an increasing trend with an increase in *v* and *δ*, respectively, and the rate of price increase gradually accelerates. This is because an increase in *v* and *δ* will lead to more pronounced market price movements, i.e., an increase in market volatility, resulting in an increase in the strike price of the variance swap. An increase in *v* and *δ* signifies an elevated probability of extreme price fluctuations. On the one hand, this augments the risk premium demanded by investors and hedgers, as they seek compensation for the heightened uncertainty. On the other hand, in markets characterized by high levels of *v* and *δ*, variance swaps become more valuable as hedging instruments, thereby elevating their strike prices. This may be attributed to the heightened demand for effective volatility protection stemming from the possibility of large and unpredictable price jumps, making variance swaps a pivotal tool for risk management in such environments.*

## 5 Conclusions

This paper focuses on pricing variance swaps within the framework of the Heston jump-diffusion model with a stochastic long-term mean. We first derive the joint moment-generating function, which is then used to derive the pricing formula for variance swaps with discrete sampling. Subsequently, to further investigate the limiting properties of the pricing formulas under discrete sampling, we also derive the pricing formula for variance swaps with continuous sampling. A series of numerical experiments are conducted to validate the accuracy and reliability of the pricing formula for discrete sampling. The numerical results computed using the discrete sampling formula are compared with Monte Carlo simulations and previous research findings. The results demonstrate a close alignment between our discrete sampling pricing formula, Monte Carlo simulation results, and existing research, confirming its effectiveness. Additionally, a thorough analysis is conducted on the specific impact of key parameters (such as *σ_θ_* and *λ*) related to the stochastic long-term mean and jump risk on the prices of variance swaps. The research findings indicate that these parameters significantly influence the prices of variance swaps, further emphasizing the importance of considering stochastic long-term mean and jump risk in pricing variance swaps.

## Supporting information

S1 Fig The comparison of prices of variance swap obtained from our formulas and obtained from MC simulations.
(EPS)

S2 Fig The impact of the key parameters of the stochastic long-term mean on the strike price of variance swaps under discrete sampling.
(EPS)

S3 Fig The impact of the key parameters of jump risk on the strike price of variance swaps under discrete sampling. (EPS)

S1 Dataset The dataset associated with S1 Fig, generated through numerical simulations conducted using MATLAB. (PDF)

S2 Dataset The dataset associated with S2 Fig, generated through numerical simulations conducted using MATLAB. (PDF)

S3 Dataset The dataset associated with S3 Fig, generated through numerical simulations conducted using MATLAB. (PDF)
