# Supplementary material for: Analytic solutions of variance swaps for Heston models with stochastic long-run mean of variance and jumps
Source: PLoS One. 2025 Mar 25;20(3):e0318886. doi: 10.1371/journal.pone.0318886 (PMC11936241; doi:10.1371/journal.pone.0318886)
Supplement: S1 Dataset — (PDF) [file pone.0318886.s004.pdf]

sample1

	Monte Carlo simulations	The discretely-sampled variance swaps	The continuously-sampled variance swaps
N=12	1145.314572	1141.076432	1133.865691
N=22	1139.882209	1137.787266	1133.865691
N=32	1138.004105	1136.558856	1133.865691
N=42	1136.794043	1135.916127	1133.865691
N=52	1136.388292	1135.521193	1133.865691
N=62	1136.20896	1135.253825	1133.865691
N=72	1135.892259	1135.060875	1133.865691
N=82	1135.567029	1134.914821	1133.865691
N=92	1135.488623	1134.800914	1133.865691
N=102	1135.194094	1134.708467	1133.865691
N=112	1134.928634	1134.633736	1133.865691
N=122	1134.818424	1134.570556	1133.865691
N=132	1134.82343	1134.517327	1133.865691
N=142	1134.599197	1134.471312	1133.865691
N=152	1134.550896	1134.431746	1133.865691
N=162	1134.545231	1134.395883	1133.865691
N=172	1134.57053	1134.365486	1133.865691
N=182	1134.543279	1134.337319	1133.865691
N=192	1134.509616	1134.313657	1133.865691
N=202	1134.493898	1134.291083	1133.865691
N=212	1134.330961	1134.270641	1133.865691
N=222	1134.510839	1134.252168	1133.865691
N=232	1134.413012	1134.236394	1133.865691
N=242	1134.356402	1134.220933	1133.865691
N=252	1134.30827	1134.205755	1133.865691
N=262	1134.225426	1134.194135	1133.865691
N=272	1134.160773	1134.18108	1133.865691
N=282	1134.185198	1134.170902	1133.865691
N=292	1134.150563	1134.158951	1133.865691
N=302	1134.126318	1134.148712	1133.865691
N=312	1134.11555	1134.14113	1133.865691
N=322	1134.35306	1134.133339	1133.865691

sample2

	Monte Carlo simulations	The discretely-sampled variance swaps	The continuously-sampled variance swaps
N=12	1288.588423	1288.997661	1280.616484
N=22	1284.630161	1285.151476	1280.616484
N=32	1283.414486	1283.724265	1280.616484
N=42	1282.514137	1282.980206	1280.616484
N=52	1282.332471	1282.523578	1280.616484
N=62	1282.298903	1282.214793	1280.616484
N=72	1282.084804	1281.992065	1280.616484
N=82	1281.846146	1281.823813	1280.616484
N=92	1281.780681	1281.692234	1280.616484
N=102	1281.531459	1281.586512	1280.616484
N=112	1281.317027	1281.49972	1280.616484
N=122	1281.224563	1281.427169	1280.616484
N=132	1281.25688	1281.365647	1280.616484
N=142	1281.072375	1281.312804	1280.616484
N=152	1281.035501	1281.266914	1280.616484
N=162	1281.031125	1281.226701	1280.616484
N=172	1281.067146	1281.191177	1280.616484
N=182	1281.039669	1281.159568	1280.616484
N=192	1281.022198	1281.131231	1280.616484
N=202	1281.024665	1281.105728	1280.616484
N=212	1280.880427	1281.082621	1280.616484
N=222	1281.032536	1281.06161	1280.616484
N=232	1280.947792	1281.042397	1280.616484
N=242	1280.918072	1281.024777	1280.616484
N=252	1280.871708	1281.008558	1280.616484
N=262	1280.814119	1280.99358	1280.616484
N=272	1280.764794	1280.979696	1280.616484
N=282	1280.777755	1280.966813	1280.616484
N=292	1280.752338	1280.954824	1280.616484
N=302	1280.736599	1280.9436	1280.616484
N=312	1280.731741	1280.933118	1280.616484
N=322	1280.913405	1280.923282	1280.616484
