# Supplementary material for: Analytic solutions of variance swaps for Heston models with stochastic long-run mean of variance and jumps
Source: PLoS One. 2025 Mar 25;20(3):e0318886. doi: 10.1371/journal.pone.0318886 (PMC11936241; doi:10.1371/journal.pone.0318886)
Supplement: S2 Dataset — (PDF) [file pone.0318886.s005.pdf]

			\kappa_{theta}		
	$\kappa_{theta}=3.2$	$\kappa_{theta}=3.3$	$\kappa_{theta}=3.4$	$\kappa_{theta}=3.5$	$\kappa_{theta}=3.6$
N=12	1284.983212	1286.050462	1287.073777	1288.055457	1288.997661
N=22	1281.158065	1282.21973	1283.237685	1284.214216	1285.151476
N=32	1279.73867	1280.798259	1281.814222	1282.788842	1283.724265
N=42	1278.998688	1280.057195	1281.07212	1282.045742	1282.980206
N=52	1278.544562	1279.602407	1280.616692	1281.589701	1282.523578
N=62	1278.23747	1279.294863	1280.308719	1281.281315	1282.214793
N=72	1278.015964	1279.073032	1280.086575	1281.058871	1281.992065
N=82	1277.848634	1278.905458	1279.918767	1280.890835	1281.823813
N=92	1277.717778	1278.774407	1279.787534	1280.759427	1281.692234
N=102	1277.612637	1278.669115	1279.682087	1280.653841	1281.586512
N=112	1277.526321	1278.582669	1279.595527	1280.567162	1281.49972
N=122	1277.454168	1278.510408	1279.523163	1280.494703	1281.427169
N=132	1277.392987	1278.449138	1279.461808	1280.433262	1281.365647
N=142	1277.340431	1278.396507	1279.409099	1280.380484	1281.312804
N=152	1277.294789	1278.3508	1279.36333	1280.33465	1281.266914
N=162	1277.2548	1278.310753	1279.323222	1280.294492	1281.226701
N=172	1277.219472	1278.275374	1279.287794	1280.259016	1281.191177
N=182	1277.188036	1278.243888	1279.256265	1280.227442	1281.159568
N=192	1277.159848	1278.215661	1279.228001	1280.199143	1281.131231
N=202	1277.134483	1278.19027	1279.202567	1280.173672	1281.105728
N=212	1277.11151	1278.167247	1279.179521	1280.150596	1281.082621
N=222	1277.09062	1278.146327	1279.158564	1280.129618	1281.06161
N=232	1277.071513	1278.127193	1279.139399	1280.110421	1281.042397
N=242	1277.05399	1278.109636	1279.121827	1280.092825	1281.024777
N=252	1277.037855	1278.093481	1279.105658	1280.07663	1281.008558

			\widetilde{\theta}		
	$\widetilde{\theta}=0.01$	$\widetilde{\theta}=0.015$	$\widetilde{\theta}=0.02$	$\widetilde{\theta}=0.025$	$\widetilde{\theta}=0.03$
N=12	615.7116602	644.8860201	674.069354	703.2616652	732.4629565
N=22	614.3235341	643.4360877	672.5535332	701.6758714	730.8031032
N=32	613.80712	642.8969393	671.9901205	701.0866641	730.1865704
N=42	613.5377009	642.6157086	671.6962772	700.7794069	729.865098
N=52	613.3723105	642.4430827	671.5159231	700.5908319	729.6678087
N=62	613.2604475	642.3263327	671.3939525	700.4633066	729.5343954
N=72	613.1797525	642.2421158	671.3059722	700.3713222	729.4381655
N=82	613.1187889	642.1784928	671.2395081	700.3018344	729.365472
N=92	613.0711112	642.1287367	671.1875306	700.2474934	729.3086247
N=102	613.0328	642.0887559	671.1457658	700.2038296	729.2629476
N=112	613.0013528	642.0559382	671.1114836	700.1679893	729.2254544
N=122	612.9750565	642.0284972	671.0828191	700.1380218	729.1941061
N=132	612.9527659	642.0052358	671.05852	700.1126185	729.1675315
N=142	612.9336194	641.9852553	671.0376481	700.0907983	729.1447055
N=152	612.9169861	641.9678982	671.0195175	700.0718439	729.1248775
N=162	612.9024121	641.9526897	671.0036313	700.0552366	729.1075049
N=172	612.889539	641.9392568	670.9895995	700.0405669	729.0921594
N=182	612.8780882	641.9273074	670.9771171	700.0275172	729.0785082
N=192	612.8678129	641.9165853	670.965917	700.0158094	729.0662609
N=202	612.858574	641.9069443	670.9558464	700.0052809	729.0552475
N=212	612.8501991	641.8982048	670.9467177	699.9957375	729.0452644
N=222	612.8425883	641.8902626	670.9384215	699.9870644	729.0361912
N=232	612.8356236	641.8829952	670.93083	699.9791286	729.02789
N=242	612.829235	641.8763293	670.9238676	699.9718497	729.0202761
N=252	612.8233575	641.8701957	670.9174606	699.9651518	729.0132699

			\sigma_{\theta}		
	$\sigma_{\theta}=0.2$	$\sigma_{\theta}=0.4$	$\sigma_{\theta}=0.6$	$\sigma_{\theta}=0.8$	$\sigma_{\theta}=1$
N=12	1290.00033	1293.00991	1298.025171	1305.048458	1314.060275
N=22	1285.699157	1287.34371	1290.082543	1293.917223	1298.825636
N=32	1284.104104	1285.244193	1287.143572	1289.801242	1293.185779
N=42	1283.268206	1284.132517	1285.572468	1287.589644	1290.12561
N=52	1282.769979	1283.50914	1284.748557	1286.471328	1288.629934
N=62	1282.422507	1283.045723	1284.086047	1285.541729	1287.343826
N=72	1282.176005	1282.729267	1283.649579	1284.938386	1286.513553
N=82	1281.980603	1282.450957	1283.243764	1284.339305	1285.66081
N=92	1281.832863	1282.254576	1282.960066	1283.945061	1285.115463
N=102	1281.704309	1282.059026	1282.652882	1283.471951	1284.436131
N=112	1281.624981	1282.001069	1282.622581	1283.514055	1284.479693
N=122	1281.523146	1281.809291	1282.293294	1282.959369	1283.658797
N=132	1281.47003	1281.782124	1282.299775	1283.0306	1283.831271
N=142	1281.427329	1281.773256	1282.34569	1283.152631	1283.999288
N=152	1281.358231	1281.631573	1282.095519	1282.723307	1283.377717
N=162	1281.312696	1281.569804	1281.998804	1282.602544	1283.15682
N=172	1281.276703	1281.531757	1281.940222	1282.552496	1283.090565
N=182	1281.262335	1281.570732	1282.07378	1282.798795	1283.501526
N=192	1281.202455	1281.41436	1281.770226	1282.265723	1282.643718
N=202	1281.188373	1281.439552	1281.859276	1282.436142	1282.930053
N=212	1281.159518	1281.388859	1281.771047	1282.313015	1282.709761
N=222	1281.153056	1281.426619	1281.879288	1282.520425	1283.081231
N=232	1281.121885	1281.359751	1281.736826	1282.298937	1282.718668
N=242	1281.093574	1281.302602	1281.646668	1282.138288	1282.43264
N=252	1281.073681	1281.267638	1281.600376	1282.055058	1282.340367

			\theta_{0}		
	$\theta_{0}=0.15$	$\theta_{0}=0.155$	$\theta_{0}=0.16$	$\theta_{0}=0.165$	$\theta_{0}=0.17$
N=12	1394.589443	1407.796652	1421.005692	1434.216564	1447.429267
N=22	1390.15087	1403.280299	1416.410731	1429.542164	1442.674598
N=32	1388.503592	1401.604106	1414.705308	1427.807198	1440.909777
N=42	1387.644718	1400.730141	1413.816089	1426.902561	1439.989557
N=52	1387.117591	1400.193748	1413.270328	1426.347331	1439.424758
N=62	1386.761117	1399.831005	1412.901248	1425.971846	1439.042799
N=72	1386.50398	1399.569345	1412.635015	1425.700991	1438.767273
N=82	1386.309732	1399.371679	1412.433895	1425.496379	1438.559131
N=92	1386.157819	1399.217093	1412.276607	1425.336359	1438.39635
N=102	1386.035758	1399.092884	1412.150225	1425.207783	1438.265556
N=112	1385.935549	1398.990911	1412.04647	1425.102225	1438.158176
N=122	1385.851783	1398.905671	1411.95974	1425.013988	1438.068417
N=132	1385.780749	1398.833387	1411.886191	1424.939161	1437.992299
N=142	1385.719735	1398.771298	1411.823016	1424.874889	1437.926917
N=152	1385.666751	1398.717381	1411.768156	1424.819076	1437.870141
N=162	1385.62032	1398.670133	1411.720082	1424.770167	1437.820387
N=172	1385.579303	1398.628393	1411.677612	1424.726959	1437.776433
N=182	1385.542803	1398.591251	1411.63982	1424.68851	1437.73732
N=192	1385.510086	1398.557959	1411.605945	1424.654047	1437.702262
N=202	1385.480638	1398.527992	1411.575454	1424.623025	1437.670705
N=212	1385.453958	1398.500841	1411.547829	1424.59492	1437.642115
N=222	1385.429696	1398.476153	1411.522708	1424.569362	1437.616116
N=232	1385.407513	1398.453579	1411.499739	1424.545995	1437.592345
N=242	1385.387167	1398.432875	1411.478673	1424.524563	1437.570543
N=252	1385.36844	1398.413818	1411.459283	1424.504836	1437.550475
