# Supplementary material for: Analytic solutions of variance swaps for Heston models with stochastic long-run mean of variance and jumps
Source: PLoS One. 2025 Mar 25;20(3):e0318886. doi: 10.1371/journal.pone.0318886 (PMC11936241; doi:10.1371/journal.pone.0318886)
Supplement: S3 Dataset — (PDF) [file pone.0318886.s006.pdf]

\lambda

	$\lambda=0.1$	$\lambda=0.2$	$\lambda=0.3$	$\lambda=0.4$	$\lambda=0.5$
N=12	1281.757338	1288.997661	1296.238444	1303.479686	1310.721388
N=22	1277.957366	1285.151476	1292.345836	1299.540444	1306.735302
N=32	1276.547678	1283.724265	1290.901022	1298.07795	1305.255049
N=42	1275.812848	1282.980206	1290.147695	1297.315313	1304.483062
N=52	1275.361916	1282.523578	1289.685345	1296.847216	1304.009193
N=62	1275.056998	1282.214793	1289.372677	1296.530648	1303.688707
N=72	1274.837066	1281.992065	1289.147139	1296.302288	1303.457514
N=82	1274.670932	1281.823813	1288.976761	1296.129775	1303.282856
N=92	1274.541011	1281.692234	1288.843517	1295.994858	1303.146259
N=102	1274.436623	1281.586512	1288.736454	1295.88645	1303.0365
N=112	1274.350928	1281.49972	1288.648562	1295.797452	1302.946391
N=122	1274.279293	1281.427169	1288.575089	1295.723054	1302.871064
N=132	1274.21855	1281.365647	1288.512786	1295.659966	1302.807187
N=142	1274.166376	1281.312804	1288.459271	1295.605776	1302.752319
N=152	1274.121067	1281.266914	1288.412797	1295.558716	1302.704671
N=162	1274.081364	1281.226701	1288.372073	1295.517478	1302.662916
N=172	1274.04629	1281.191177	1288.336096	1295.481047	1302.626029
N=182	1274.015081	1281.159568	1288.304084	1295.44863	1302.593207
N=192	1273.987104	1281.131231	1288.275386	1295.41957	1302.563782
N=202	1273.961925	1281.105728	1288.249559	1295.393416	1302.5373
N=212	1273.93911	1281.082621	1288.226156	1295.369718	1302.513305
N=222	1273.918367	1281.06161	1288.204878	1295.34817	1302.491487
N=232	1273.899398	1281.042397	1288.18542	1295.328466	1302.471536
N=242	1273.882001	1281.024777	1288.167575	1295.310395	1302.453239
N=252	1273.865988	1281.008558	1288.151149	1295.293762	1302.436396

\eta

	$\eta=0.02$	$\eta=0.04$	$\eta=0.06$	$\eta=0.08$	$\eta=0.1$
N=12	1280.923446	1286.303012	1291.695536	1297.100898	1302.518981
N=22	1277.125725	1282.471858	1287.835428	1293.216227	1298.61405
N=32	1275.716796	1281.050278	1286.403039	1291.774831	1297.165408
N=42	1274.982344	1280.30917	1285.656278	1291.023397	1296.410257
N=52	1274.531639	1279.854358	1285.19799	1290.562248	1295.946848
N=62	1274.226871	1279.546803	1284.888083	1290.25041	1295.633491
N=72	1274.007046	1279.324963	1284.664543	1290.025479	1295.407469
N=82	1273.840992	1279.157384	1284.495679	1289.855565	1295.236734
N=92	1273.711133	1279.026331	1284.363621	1289.722687	1295.103215
N=102	1273.606794	1278.921031	1284.257514	1289.61592	1294.995934
N=112	1273.52114	1278.834587	1284.170406	1289.528272	1294.907865
N=122	1273.449539	1278.762325	1284.09759	1289.455004	1294.834245
N=132	1273.388824	1278.70105	1284.035844	1289.392875	1294.771819
N=142	1273.336675	1278.648419	1283.982809	1289.339511	1294.718198
N=152	1273.291387	1278.602713	1283.936751	1289.293168	1294.671633
N=162	1273.251702	1278.562661	1283.896392	1289.252559	1294.63083
N=172	1273.216645	1278.52728	1283.860739	1289.216684	1294.594784
N=182	1273.185451	1278.495797	1283.829014	1289.184763	1294.56271
N=192	1273.157486	1278.467573	1283.800573	1289.156146	1294.533957
N=202	1273.132319	1278.442173	1283.774978	1289.130392	1294.50808
N=212	1273.109515	1278.419158	1283.751786	1289.107056	1294.484633
N=222	1273.088781	1278.398232	1283.730699	1289.085839	1294.463314
N=232	1273.069821	1278.379096	1283.711416	1289.066436	1294.443819
N=242	1273.052432	1278.361546	1283.693731	1289.048642	1294.42594
N=252	1273.036426	1278.345393	1283.677453	1289.032263	1294.409483

v

	$v=0.01$	$v=0.02$	$v=0.03$	$v=0.04$	$v=0.05$
N=12	1288.997661	1289.537044	1290.502926	1291.90811	1293.765706
N=22	1285.151476	1285.65809	1286.588215	1287.954566	1289.770158
N=32	1283.724265	1284.217454	1285.133015	1286.483628	1288.282274
N=42	1282.980206	1283.466094	1284.373752	1285.715842	1287.505327
N=52	1282.523578	1283.004877	1283.907573	1285.244319	1287.028065
N=62	1282.214793	1282.692941	1283.592234	1284.925316	1286.70513
N=72	1281.992065	1282.467915	1283.364727	1284.69514	1286.472091
N=82	1281.823813	1282.297914	1283.192838	1284.521221	1286.295995
N=92	1281.692234	1282.164959	1283.058398	1284.385184	1286.158246
N=102	1281.586512	1282.058126	1282.950366	1284.275863	1286.047544
N=112	1281.49972	1281.970418	1282.861671	1284.186106	1285.95665
N=122	1281.427169	1281.897098	1282.787523	1284.111069	1285.880658
N=132	1281.365647	1281.834924	1282.724644	1284.047433	1285.816212

N=142	1281.312804	1281.781518	1282.670632	1283.99277	1285.760851
N=152	1281.266914	1281.735139	1282.623726	1283.945297	1285.712771
N=162	1281.226701	1281.694497	1282.582621	1283.903694	1285.670636
N=172	1281.191177	1281.658593	1282.546307	1283.866941	1285.633411
N=182	1281.159568	1281.626645	1282.513994	1283.834235	1285.600286
N=192	1281.131231	1281.598004	1282.485026	1283.804916	1285.570589
N=202	1281.105728	1281.572227	1282.458954	1283.778527	1285.543861
N=212	1281.082621	1281.548871	1282.43533	1283.754616	1285.519642
N=222	1281.06161	1281.527635	1282.41385	1283.732874	1285.49762
N=232	1281.042397	1281.508215	1282.394208	1283.712992	1285.477482
N=242	1281.024777	1281.490404	1282.376193	1283.694758	1285.459012
N=252	1281.008558	1281.474011	1282.359611	1283.677973	1285.442011

			\delta		
	$\delta=0.02$	$\delta=0.04$	$\delta=0.06$	$\delta=0.08$	$\delta=0.1$
N=12	1288.997661	1291.486693	1295.658293	1301.547499	1309.203813
N=22	1285.151476	1287.623047	1291.765343	1297.613102	1305.215386
N=32	1283.724265	1286.18917	1290.320283	1296.152225	1303.733896
N=42	1282.980206	1285.441593	1289.566801	1295.390391	1302.961179
N=52	1282.523578	1284.982789	1289.104346	1294.922774	1302.486834
N=62	1282.214793	1284.672526	1288.791603	1294.606523	1302.166013
N=72	1281.992065	1284.448727	1288.566009	1294.37839	1301.934572
N=82	1281.823813	1284.279665	1288.395587	1294.206046	1301.759723
N=92	1281.692234	1284.147451	1288.262308	1294.071261	1301.622974
N=102	1281.586512	1284.041218	1288.155218	1293.962958	1301.513092
N=112	1281.49972	1283.954007	1288.067302	1293.874045	1301.422881
N=122	1281.427169	1283.881104	1287.993809	1293.799719	1301.347468
N=132	1281.365647	1283.819284	1287.931489	1293.73669	1301.283518
N=142	1281.312804	1283.766184	1287.877959	1293.682552	1301.228587
N=152	1281.266914	1283.720071	1287.831472	1293.635537	1301.180883
N=162	1281.226701	1283.679663	1287.790736	1293.594338	1301.13908
N=172	1281.191177	1283.643967	1287.75475	1293.557942	1301.10215
N=182	1281.159568	1283.612203	1287.722729	1293.525556	1301.069288
N=192	1281.131231	1283.583728	1287.694023	1293.496523	1301.03983
N=202	1281.105728	1283.558102	1287.668188	1293.470393	1301.013316
N=212	1281.082621	1283.534882	1287.644779	1293.446717	1300.989293
N=222	1281.06161	1283.513769	1287.623494	1293.42519	1300.967449
N=232	1281.042397	1283.494462	1287.604031	1293.405505	1300.947474
N=242	1281.024777	1283.476756	1287.58618	1293.387451	1300.929155
N=252	1281.008558	1283.460458	1287.56975	1293.370833	1300.912293
