# Supplementary material for: Analytic solutions of variance swaps for Heston models with stochastic long-run mean of variance and jumps
Source: PLoS One. 2025 Mar 25;20(3):e0318886. doi: 10.1371/journal.pone.0318886 (PMC11936241; doi:10.1371/journal.pone.0318886)
Supplement: Attachment — Submitted filename: Response to Reviewers.pdf [file pone.0318886.s007.pdf]

Dear Editor:

Thank you for your letter and for two reviewers' comments concerning our manuscript ID PONE-D-24-55815. Those comments are all valuable and very helpful for revising and improving our paper, as well as the important guiding significance to our research. We have studied the comments carefully and made a correction that we hope meets with approval. Revised portions are marked in blue on the paper. The main corrections in the paper and the responses to the reviewer's comments are as follows.

Reply to Academic editor

Q1. Please ensure that your manuscript meets PLOS ONE's style requirements, including those for file naming.

Answer: Thank you very much for your careful review and valuable suggestions regarding our manuscript. I have thoroughly revised the manuscript according to PLOS ONE's style requirements, using the template files you provided as reference. All aspects including file naming, main text formatting, title page, author information and affiliations have been properly adjusted. To ensure full compliance with the journal's requirements, I have meticulously checked the following elements:File naming conventions, Title page format, Manuscript structure, Reference formatting, Figure and table legends. I am attaching the revised manuscript files for your kind review. Should there be any additional modifications required, I would be more than happy to make the necessary changes promptly.

Q2. Please note that PLOS ONE has specific guidelines on code sharing for submissions in which author-generated code underpins the findings in the manuscript.

Answer: In accordance with your guidelines, we are pleased to confirm that we will make all author-generated code underlying the findings in our manuscript publicly available upon publication. To facilitate access, we will include the following statement in the

manuscript:"The code supporting the findings of this study is available from the corresponding author upon reasonable request. Readers may contact [1200202z1009@smail.swufe.edu.cn] to request access. "We believe this approach will ensure compliance with PLOS ONE's policies while maintaining the necessary safeguards for our work. For any further guidance or specific requirements regarding code sharing, I am at your disposal to implement them without delay.

Q3. Please confirm at this time whether or not your submission contains all raw data required to replicate the results of your study. Authors must share the "minimal data set" for their submission.

Answer: The complete dataset has been compiled in an Excel file titled "Data." This file has been uploaded as part of our Supporting Information submission and is readily accessible for review. We believe this dataset fulfills PLOS ONE's requirements for the "minimal data set" as defined in your guidelines, as it contains all information necessary to reproduce the findings reported in our manuscript. I welcome any additional queries or requests for clarification regarding the dataset and will respond to them with utmost priority.

Q4. Please confirm at this time whether or not your submission contains all raw data required to replicate the results of your study. Authors must share the "minimal data set" for their submission.

Answer: We have carefully checked the reference list to ensure its completeness and accuracy. After reviewing all cited papers, we confirm that none of the references included in our manuscript have been retracted.Your continued feedback is invaluable to us, and I remain fully committed to addressing any additional points or suggestions you may have during the review process.

Reply to Reviewer 1

Q1. I suggest the author uses Origin or OriginPro for scientific graphing to

make the presented graphs more clear.

Answer: We are extremely grateful for your meticulous review and invaluable suggestions on our manuscript. We feel deeply honored and attach great importance to the feedback you have provided, as it has played a crucial role in revising and improving our paper. Regarding the issue of graph clarity you mentioned, we have re-run the corresponding programs using MATLAB software and successfully generated clearer and more easily identifiable images. We ensure that these images can more accurately convey our research data and results. In our future studies and research, we will actively refer to and adopt your suggestions, delve deeper into the use of Origin software, and strive to apply it in our subsequent research work to further enhance the quality and readability of our paper's charts and graphs. Once again, thank you for your thoughtful guidance and valuable opinions. We look forward to continuing to make progress and improvements in our research with your help.

Reply to Reviewer 2

Q1. Expanded sensitivity analyses and discussions on computational efficiency.

Answer: Thank you very much for your meticulous review and valuable suggestions on our manuscript. I have made corresponding adjustments based on your comments. Firstly, regarding the extended sensitivity analysis, I have made the following modifications to Figures 2 and 3. Specifically, From Figure 2, we observe that the primary parameters of the stochastic long-term mean significantly impact the strike price of the variance swap. Specifically, the strike price of the variance swap increases with an increase in κ_θ. This is because κ_θ determines the speed at which the long-term mean θ_t reverts to its long-term average $\tilde{\theta}$. Specifically, when κ_θ is high, θ_t converges to $\tilde{\theta}$ more rapidly. Over the life of the contract, this leads to an increase in the variability of v_t, which in turn amplifies the

fluctuations in realized returns, thereby raising the fair strike price of the variance swap. Conversely, when κ_θ is low, the reversion of θ_t to $\tilde{\theta}$ occurs more slowly. Over the contract period, the variability of v_t becomes relatively smaller, resulting in reduced fluctuations in realized returns and, consequently, a lower fair strike price for the variance swap. The strike price of the variance swap exhibits an increasing trend with an increase in $\tilde{\theta}$ and θ_0, and the increase occurs at a relatively steady rate. This is because when $\tilde{\theta}$ is high, θ_t tends to fluctuate around a higher level, thereby pulling v_t toward higher values. This increases the variability of $\frac{S_{t_i}-S_{t_{i-1}}}{S_{t_i}}$, leading to a higher realized variance and, consequently, a higher fair strike price. In markets with low volatility expectations (e.g., during periods of economic stability or calm market sentiment), investors demand lower risk premiums. As a result, the fair strike price of variance swaps decreases. Additionally, if the market's expectation of future volatility is high at the initial time, the volatility of asset prices will be greater in the early stages of the contract, resulting in higher realized variance and, consequently, an increase in the fair strike price of the variance swap. Furthermore, the strike price of the variance swap increases with an increase in σ_θ, and the rate of increase gradually decreases with an increase in the sampling frequency. The increase in σ_θ leads to a higher strike price for variance swaps due to the greater uncertainty and instability it introduces into the long-term volatility dynamics. This uncertainty increases the risk premium demanded by market participants, driving up the price of variance swaps. However, as sampling frequency increases, the rate at which the strike price rises slows down, reflecting the averaging effect of more frequent measurements.

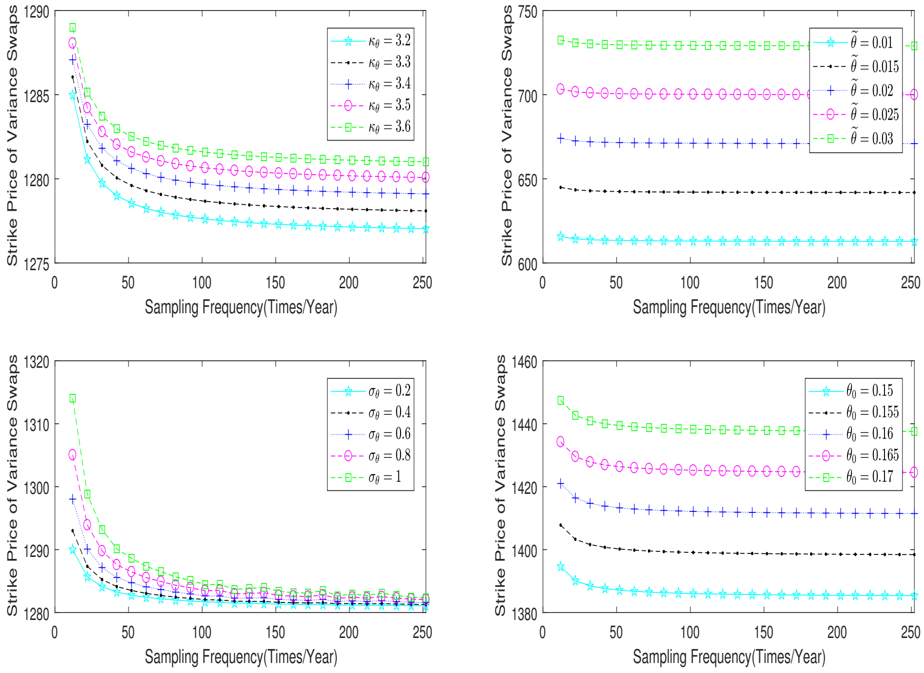

Figure 2: The impact of the key parameters of the stochastic long-term mean on the strike price of variance swaps under discrete sampling.

Observing Figure 3 reveals that the key parameters related to the jump component play a crucial role in determining the strike price of the variance swap. Specifically, the variance swap shows a high sensitivity to λ, with the strike price gradually increasing as λ increases. Specifically, the jump intensity λ reflects the probability of price jumps in the underlying asset within a unit of time. A higher jump intensity implies a greater likelihood of significant short-term fluctuations in the underlying asset price, indicating an expected increase in future volatility, leading to an increase in the price of the variance swap. A higher λ increases the uncertainty about future asset price movements. Investors and hedgers demand greater compensation for this heightened uncertainty, thereby leading to an increase in the fair strike price of the variance swap. The strike price of the variance swap gradually increases with an increase in η. This is because an increase in η will increase the volatility of the variance, leading to an increase in the volatility of the underlying asset price, thereby increasing the strike price of the variance swap. This increased variability

in volatility translates to higher uncertainty about future market conditions. As a result, the strike price of the variance swap rises to reflect the heightened risk. The strike price of the variance swap shows an increasing trend with an increase in v and δ, respectively, and the rate of price increase gradually accelerates. This is because an increase in v and δ will lead to more pronounced market price movements, i.e., an increase in market volatility, resulting in an increase in the strike price of the variance swap. An increase in v and δ signifies an elevated probability of extreme price fluctuations. On the one hand, this augments the risk premium demanded by investors and hedgers, as they seek compensation for the heightened uncertainty. On the other hand, in markets characterized by high levels of v and δ, variance swaps become more valuable as hedging instruments, thereby elevating their strike prices. This may be attributed to the heightened demand for effective volatility protection stemming from the possibility of large and unpredictable price jumps, making variance swaps a pivotal tool for risk management in such environments.

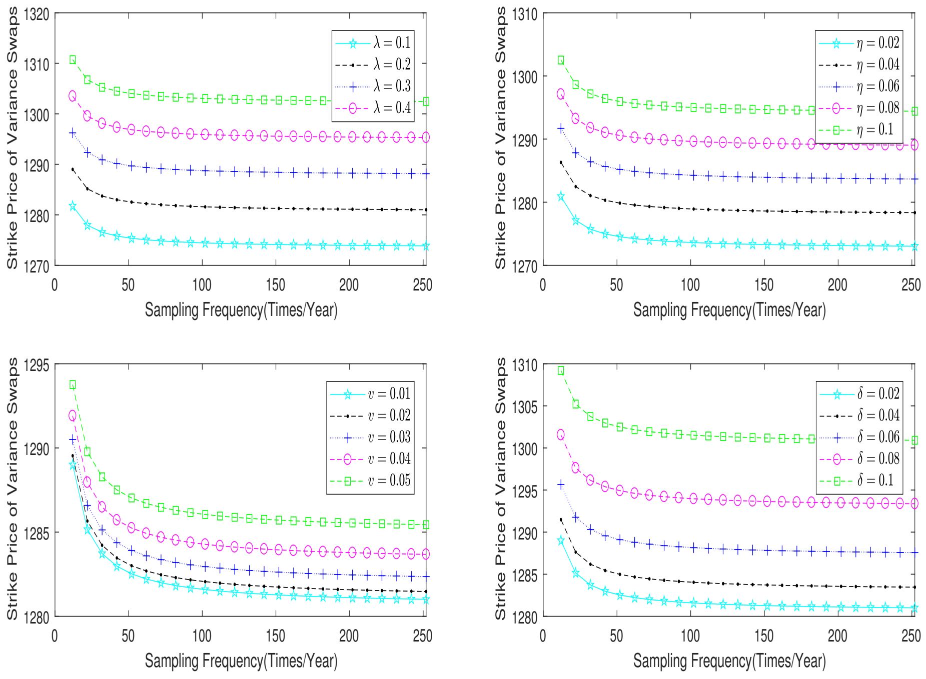

Figure 3: The impact of the key parameters of junp risk on the strike price of variance swaps under discrete sampling.

Secondly, concerning the discussion on computational efficiency, I have added the corresponding CPU computation times in Table 2. The calculations were performed on an Intel(R) Core(TM) i5-12500H. Specifically, We exhibit a comparison between the numerical implementation of Eq(15) and the Monte Carlo (MC) simulation results in Figures 1 and Table 2. Figure 1 displays three sets of data: one set represents the variance swap prices derived from 500,000 Monte Carlo simulations based on Eq(14), another set is calculated using Eq(15) for discretely-sampled variance swaps, and the third set is obtained from Eq(18) for continuously-sampled variance swaps. In Table 2, we present a detailed listing of the numerical results for variance swaps under four different sampling frequencies along with their corresponding relative errors. In this table, AS denotes the discretely-sampled analytic solution, MC represents the Monte Carlo simulation results, CON stands for the continuously-sampled analytic solution, and RD signifies the standard error. Additionally, we have recorded the CPU runtime to facilitate a comprehensive comparison of the computational efficiency of the two methods. All calculations were performed on an Intel(R) Core(TM) i5-12500H processor. It is evident from the table that the analytic formula can produce highly accurate results within an extremely short period of time. Notably, as the sampling frequency increases, the time required to compute the fair strike price of variance swaps using Monte Carlo simulation becomes significantly longer than that required using our proposed analytic pricing formula. From Figure 1 and Table 2, we observe that as the sampling frequency increases, the strike price of the variance swap under discrete sampling gradually converges to that under continuous sampling. Furthermore, with increasing sampling frequency, the strike price of the variance swap under discrete sampling becomes very close to the price obtained through MC simulations, this indicates that the results obtained from our analytical results in Theorem1 are in high agreement with those from the MC simulations.

Table 2: **Fair strike prices K_{var} derived from MC simulation, the analytic formula and the continuous model, along with their corresponding relative errors.**

**Sample**	N	**AS**	**MC**	**CON**	**RD**
Sample 1	Monthly ($N = 12$)	1141.0764	1145.0146	1133.8657	0.3439
	CPU time (s)	0.0115	1.1463	-	-
	Fortnightly ($N = 26$)	1137.1822	1138.5908	1133.8657	0.1237
	CPU time (s)	0.0141	1.6675	-	-
	Weekly ($N = 52$)	1135.5212	1136.1883	1133.8657	0.0587
	CPU time (s)	0.01185	2.7977	-	-
	Daily ($N = 252$)	1134.2058	1134.3083	1133.8657	0.0090
	CPU time (s)	0.0147	11.4784	-	-
Sample 2	Monthly ($N = 12$)	1288.9977	1288.5884	1280.6165	-0.0318
	CPU time (s)	0.0071	1.1323	-	-
	Fortnightly ($N = 26$)	1284.4478	1283.9812	1280.6165	-0.0363
	CPU time (s)	0.0087	1.8132	-	-
	Weekly ($N = 52$)	1282.5236	1282.3325	1280.6165	-0.0149
	CPU time (s)	0.0092	3.6321	-	-
	Daily ($N = 252$)	1281.0086	1280.8717	1280.6165	-0.0107
	CPU time (s)	0.0136	10.6012	-	-

Table notes: We provide numerical results for variance swaps under three different sampling frequencies, along with their corresponding relative errors. In this table, AS, MC, CON and RD represent the discretely-sampled analytic solution, Monte Carlo simulation results, continuously-sampled analytic solution and standard error, respectively.
